# Active Transport of Peptides Across the Intact Human Tympanic Membrane

**DOI:** 10.1038/s41598-018-30031-6

**Published:** 2018-08-07

**Authors:** Arwa Kurabi, Daniel Schaerer, Volker Noack, Marlen Bernhardt, Kwang Pak, Thomas Alexander, Jacob Husseman, Quyen Nguyen, Jeffrey P. Harris, Allen F. Ryan

**Affiliations:** 1 University of California, San Diego, Division of Otolaryngology, Department of Surgery, La Jolla, CA 92093 USA; 2 San Diego Veterans Affairs Healthcare System, Research Department, San Diego, CA 92130 USA; 3 Ruhr-Universitat Bochum, Department of ENT, Bochum, NRW Germany; 4 Universitätsklinik Würzburg, Department of ENT, Würzburg, 97070 Germany

## Abstract

We previously identified peptides that are actively transported across the intact tympanic membrane (TM) of rats with infected middle ears. To assess the possibility that this transport would also occur across the human TM, we first developed and validated an assay to evaluate transport *in vitro* using fragments of the TM. Using this assay, we demonstrated the ability of phage bearing a TM-transiting peptide to cross freshly dissected TM fragments from infected rats or from uninfected rats, guinea pigs and rabbits. We then evaluated transport across fragments of the human TM that were discarded during otologic surgery. Human trans-TM transport was similar to that seen in the animal species. Finally, we found that free peptide, unconnected to phage, was transported across the TM at a rate comparable to that seen for peptide-bearing phage. These studies provide evidence supporting the concept of peptide-mediated drug delivery across the intact TM and into the middle ears of patients.

## Introduction

Otitis media (OM), the most common pediatric infectious disease requiring treatment, causes more office visits and drug purchases than any other condition in young children: more than 90% of children experience OM before age 5^1,2^, while recurrent (>6 episodes) and chronic OM affect approximately 20%^3,4^. OM is also the most common reason for pediatric surgery requiring general anesthesia; resulting in more than 700,000 US procedures in 2008^5^. While streptococcal vaccines have reduced OM due to the covered strains, incidence has increased for other strains and microbes, thus the overall decrease in disease burden is relatively modest^6,7^. OM is estimated to cost the US nearly $3 billion/year for health care^8^, and an additional $3 billion/year in associated costs^9,10^.

Persistent OM produces conductive hearing loss during critical periods of language acquisition^11^ and may result in fibrosis and scarring that permanently damage the conductive apparatus of the middle ear (ME): 15–20% of teenagers exhibit visible tympanic membrane (TM) scarring due to prior OM^12^. Severe OM may also damage the inner ear, due to transit of injurious substances through the round window membrane, resulting in permanent hearing loss^13^. Although the long-term impacts of OM are controversial, hearing loss due to OM has been linked to speech perception deficits^14^, language delay^15,16^ and learning disabilities^17,18^. OM has far more serious consequences in some countries of the developing world. Worldwide, OM causes an estimated 28,000 annual deaths and contributes to more than 175 million cases of serious hearing loss, greater than half the world burden of significant hearing impairment^19,20^. For the countries in which this is the case, much of the population lacks access to adequate primary health care, and to ENT surgeons who can diagnose and provide advanced treatment for more serious OM.

Systemic antibiotics are no longer recommended for uncomplicated acute OM (AOM), or OM with effusion in older children^21,22^, although treatment patterns have changed relatively little since this recommendation^23^. Antibiotics are recommended for children under age two, and for more complicated cases^24^. Presumably because of widespread oral antibiotic usage, the incidence of resistant microbes recovered from the MEs of OM patients is increasing^6,25^. Access of systemically delivered antibiotics to the ME is also a concern. About 20% of children receiving orally administrated antibiotics for OM show no detectable levels in the tympanic cavity^26^. Ventilation tubes can be effective for recurrent or chronic OM (e.g. MRC Multicentre OM Study Group, 2004)^27^, but there is controversy about the long-term benefit and the potential for later TM abnormalities^28–30^.

Given the above, there is a clear need for alternative OM therapies. Local delivery of antibiotics would decrease off-target exposure of pathogens and ensure bactericidal levels in the ME. However, the impermeable TM makes this route considerably challenging unless the membrane is breached. Using the technique of phage display, we recently discovered unique peptides that can transport large (1 μm in length and 7 nm in diameter) phage particles across the intact TM of rats undergoing bacterial OM^31^. This active transport could form the basis for local drug delivery to the ME without the need for surgery or injections through the TM, both of which require general anesthesia in children. However, a number of questions remain to be answered about trans-TM transport. Does transport occur in other species, or is it limited to the rat? Is transport limited to the infected TM, or might it occur in normal membranes? Does transport depend upon linkage of peptide to bacteriophage, or is the peptide alone sufficient to mediate transport? To address these issues, we developed an *in vitro* chamber assay system, in which fragments of the TM from various species can be tested before proceeding to human translational work. This was used to determine the rate of peptide phage transport observed for the rat TM with that across the TMs isolated from guinea pigs, rabbits and humans postmortem.

## Materials and Methods

### Subjects

Subjects were: (1) Sprague Dawley rats 250–350 g (60–90 days old); (2) Hartley guinea pigs 200–350 g; and (3) New Zealand white rabbits, 1–2 kg. All experiments were performed to National Institutes of Health guidelines and approved by the Institutional Animal Care and Use Committee of the VA San Diego Medical Center. Excised fragments of the human TM were obtained as wastage from reconstructive and other otologic surgeries at University of California, San Diego (UCSD) Medical Centers. The use of human tissues was reviewed by the Institutional Review Board (IRB) of the UCSD School of Medicine. It was determined that use of unidentifiable and wastage TM pieces removed during ear surgery was not considered human research and did not need patient consent as these pieces are normally discarded tissue and are not sent to the department of pathology for diagnoses or further evaluation. All experiments were performed in accordance with the relevant guidelines and regulations outline by the UCSD Human Research Protections Program IRB standards.

### TM harvest

Animals were deeply anesthetized with an appropriate anesthetic (rodent, guinea pig or rabbit cocktail, I.M.) and sacrificed. The ME bullae were dissected from the cranium and the TM widely exposed by enlarging the external auditory canal and opening the ME cavity medially. The TM was carefully separated from the annulus and the manubrium of the malleus with a No. 11 scalpel blade, being careful not to perforate the membrane. The membranes were either used immediately or placed on cold PBS on ice until use. Human TM samples discarded during otologic surgery were immediately immersed in cold PBS and placed on ice for transport to the laboratory. All TM fragments were used in the *in vitro* assay within 0.5–2 hours of harvest.

### Bacteriophage and free peptide

Bacteriophage had been isolated previously by screening a NEB 12-mer library of phage engineered to express 10^10^ random 12-mer peptides on their surfaces, integrated onto the C-terminus of the M13 phage pIII surface protein. Peptides were linked to pIII with a short GGG spacer. Peptide-bearing phage were applied to the TMs of rats in which OM had been induced by the injection of strain 3566 nontypeable *Haemophilus influenza* (NTHi) through the bone of the ventral bulla, taking care not to damage the TM. This resulted in the isolation of several peptides that could actively transit the TM^31^. The most promising candidate of these 12-mer peptides, in terms of rate of transport, was chosen for further study *in vitro*. In order to test the ability of peptide to transit the TM without being linked to bacteriophage, this peptide was synthesized and conjugated to a DNA PCR template (Bio-synthesis) for quantitative detection.

### TM *in vitro* exposure chambers

The goal of this study was to develop and carefully characterize an *in vitro* model system that could utilize human TM fragments isolated during otologic surgeries, to determine whether active, peptide-mediated transtympanic transport occurs in a similar manner to that previously observed in the rat^31^. Our previous study established that transport occurs *ex vivo* for up to six hours post-mortem, providing the basis for an *in vitro* approach.

Our assay system consists of two cylindrical chambers, separated by a silicon membrane gasket with an appropriately sized opening. The upper chamber, representing the external ear, is filled with fluid containing peptide-bearing phage or free peptide for varying periods of time. The lower chamber represents the middle ear lumen. A TM fragment appropriately sealed by the gasket completes the model system.

**Figure 1:**
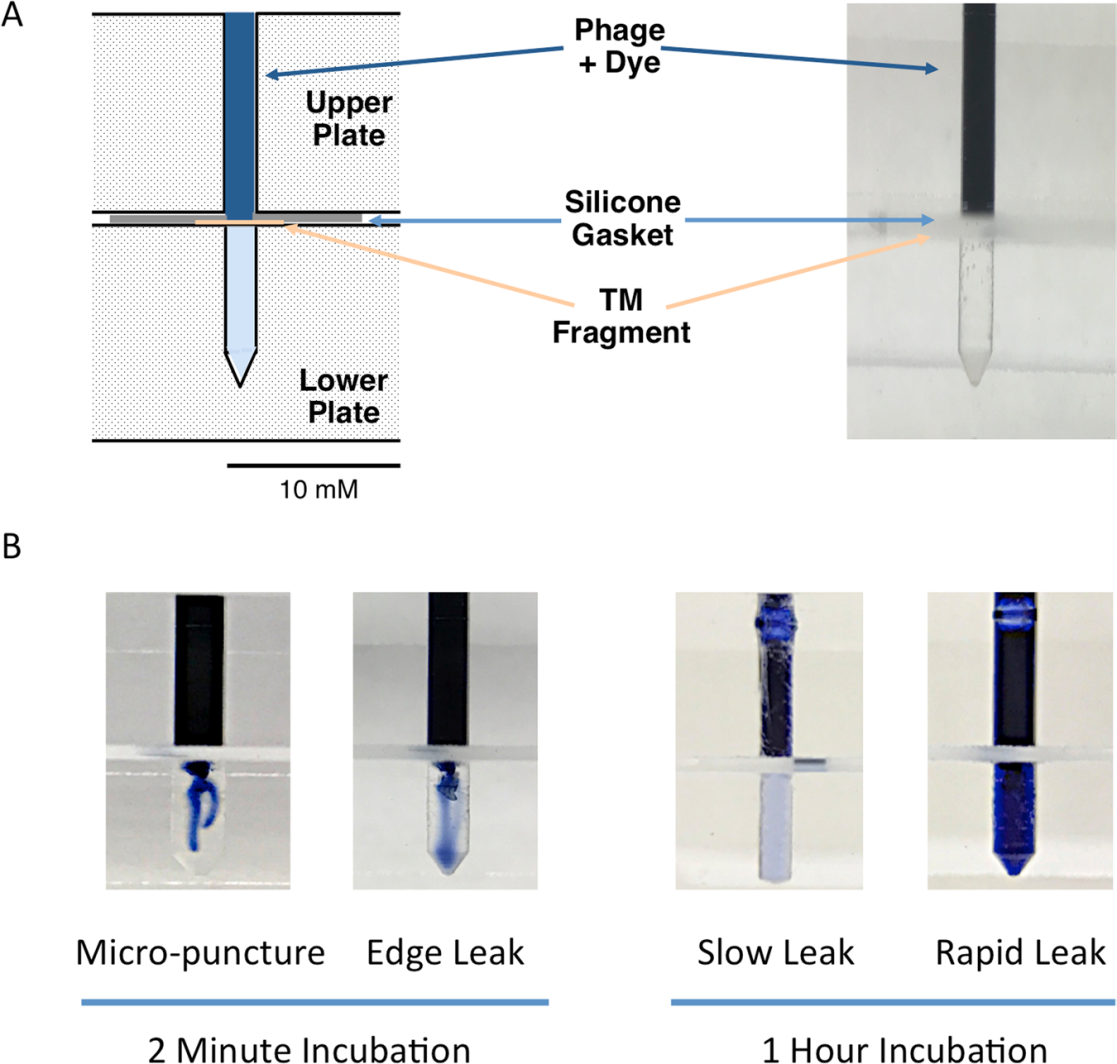
Schematic diagram and photographic images of the modified Ussing chamber assay for transport across the TM. (**A**) The chambers were fabricated from two, stacked layers of 12 mm Lucite plastic. In the bottom layer, a series of 8 mm deep wells formed the lower wells. In the upper layer, an identically sized and place series of holes penetrated the thickness of the plastic, forming the upper well. Pieces of Silastic rubber sheeting 0.5 mm thick and with holes corresponding to the well diameters formed the gasket sealer. Additional holes through the device allowed the upper and lower plates to be compressed by means of inserted stainless-steel screws, washers and wing nuts. Transport across the TM was detected by evaluating the phage titers in the fluid of the lower chamber. (**B**) Leakage of fluid through the TM caused by incomplete seal of the TM fragment edge or damage is immediately apparent. However, small leaks are detected colorimetrically at the end of the incubation period.

The assay design is illustrated in Fig. 1A. The upper and lower chambers were fabricated from two layers of 12 mm Lucite (Dow Chemical) plastic. In the lower layer, a series of wells was created, 8 mm deep and ranging in diameter from 1.0 to 3.0 mm, forming the lower chambers. In the upper layer, an identically sized and place series of holes penetrated the thickness of the plastic, forming the upper chambers. Silastic (Dow Chemical) rubber sheeting 0.5 mm thick and with holes corresponding in size and placement to the Lucite wells was used for the gaskets. Additional holes through the device and gasket accommodated stainless steel screws that could be tightened with washers and wing nuts, sealing a TM fragment into place between the two chambers.

### TM exposure to peptide phage or free peptide

Each TM was divided and the lower assay chamber was filled with PBS. A TM fragment was placed over the opening of the lower chamber, sized to allow the edges to overlap the plastic by at least 1 mm around the edge of the opening. The Silastic gasket was then placed over the TM, with the hole aligned to the lower chamber, the second plate was applied, and the devise compressed to seal the TM fragment into place. The upper opening was then filled with the 10^9^ peptide-bearing bacteriophage or 1 μM of DNA-tagged free peptide. TM exposure to phage or peptide varied from 1 to 4 hours, and was performed at room temperature. Controls consisted of an identical concentration of wild-type (WT) phage (not bearing a peptide) or the DNA tag alone.

At the end of each exposure, the upper chamber was carefully washed with glycine/salt acid wash for 15 min and then washed 4x with PBS to remove any excess phage or peptide from the upper chamber, and the PBS removed. The device was then disassembled, the gasket and TM fragment removed, and fluid recovered from the lower chamber for analysis.

### Phage titer determination

To quantify phage titers prior to use in the assay, 10 μL various dilutions of the desired phage clone were mixed with 200 μl of *E.coli* ER2738 and 3 mL of melted top agar, vortexed briefly, and poured immediately onto plates containing LB, isopropyl beta-D-thiogalactopyranoside (IPTG), and 5-bromo-4-chloro-3-indolyl-β-D-galactopyranoside (X-gal**)**. Plates were incubated at 37 °C overnight and the blue plaques were visualized, counted and multiplied by appropriate dilution factors to determine the plaque forming units (PFUs) per μL. To assess phage transit of TM fragments in the assay or *in vivo*, 1 μL of each ME or lower chamber sample was serially diluted, mixed with *E*. *coli* host strain and liquid top agar, applied to LB/PTG/X-gal plates, incubated, counted as above. Counts were multiplied by the appropriate dilution factors to convert titer results into plaque-forming units (PFUs) per total volume of the lower chamber.

### Integrity of the TM fragment and seal

A central issue for this *in vitro* assay was the integrity of the TM fragments and the seal between the two chambers. Leakage could be caused by damage to the TM during dissection producing a defect in the membrane, or by an inadequate seal around the edge of the fragment. To determine whether there was leakage between the upper and lower chambers when a TM fragment was in place, we used three methods. In the first, we inserted wires into the two chambers and measured the resistance across the TM fragment. This method proved unreliable, due to excessive variability in resistance. This variability was such that micropunctures 10–50 μm in diameter, made in the center of the TM fragment with the end of a glass pipette, could not be reliably detected. We noted similar variability when resistance was measured between the fluid-filled rat external ear canal and the fluid-filled ME, across the intact TM *in situ*. This method was then abandoned.

For the second method, we added 0.5% trypan-blue to the upper chamber (Fig. 1B) and assessed dye transit into the lower chamber, immediately and after 1–4 hours, visually and colorimetrically using a Nanodrop 2000 UV-VIS spectrophotometer (Thermofisher) and setting the absorbance wavelength to 590 nm. To assess leak detection, TM fragments were deliberately placed in the chamber with an incomplete seal on the edge. Other TMs were penetrated with a glass micropipette to create openings of various sizes. In addition, small unintentional leaks were sometimes observed in presumably intact and correctly placed TMs.

The third method of leak detection was to place a wild-type (WT) phage, which did not express a peptide, into the upper chamber along with trypan-blue dye as above. Rat TMs were interposed between the two chambers. Since WT phage are unable to cross the intact TM *in vivo*^31,32^, any phage detected in the lower chamber would represent leakage or contamination. Micropunctures of various sizes were created in the TM as above. After 1 hour, the fluid in the lower chamber was titered. The WT phage titers were then compared to dye levels.

### Peptide detection by qPCR

The lead peptide TMT-3 (SADSTKTTHLTL) identified through the phage screen^31^ was synthesized by Bio-synthesis (TX, USA). The synthetic peptide was attached to a 156 bp DNA tag (5′-GGAGAAGAACTTTTCACTGGAGTTGTCCCAATTCTTGTTGAATTAGATGGTGATGTTAATGGGCACAAATTTTCTGTCAGTGGAGAGGGTGAAGGTGATGCAACATACGGAAAACTTACCCTTAAATTTATTTGCACTACTGGAAAACTACCTGTT-3′ which served as a DNA-template for qPCR detection and quantitation. The peptide was suspended in PBS so that the molar concentration matched that of the peptide phage as used above (1 μM). The DNA tag alone (without the peptide conjugate) served as an experimental control. The unconjugated DNA tag was applied at the same molar concentration of 1 μM. The assay device was then assembled as above with PBS in the lower chamber and PBS plus tagged peptide in the upper chamber. Following incubation for 1 hr. or 4 hrs., the upper chamber was carefully washed. The apparatus was then disassembled and the fluid in the lower chamber was collected. One microliter served as a template for real-time qPCR amplification. Reactions were run on an Applied Biosystems thermal cycler Real-Time PCR with SYBR green detection (AB SYBR Green 2 × master mix) with the following parameters: 10 min at 95 °C; 45 × [95 °C for 45 sec, 60 °C for 45 sec,] 72 °C for 2 min. The amount of internalized peptide was determined using a standard curve method (i.e. Linear regression of Ct vs. concentration of DNA standards generated from above DNA template). This relationship was used to calculate the concentration of DNA in present in the sample based on the Ct values. Efficiency of primers was determined to be 95–100% by standard curve, and melt curves and agarose gel detection were used to assure the correct amplicon size of 156 bp.

## Results

### Integrity of the TM fragment

When trypan-blue was added to the upper assay device chamber, the dye was excluded from the lower chamber by an intact TM fragment. However, the presence of dye in the lower chamber proved able to reliably detect micropunctures in the TM. In this case, dye was initially detected at the site of the TM puncture. Deliberate or unintentional leaks due to an incomplete seal of the edge of a TM fragment were also detected, initially visible at the edge of the chamber. In some cases of micropuncture, the defect sealed itself after withdrawal of the micropipette tip, leaving a defect too small to be seen. In the case of slow leaks caused by these extremely small defects created in the TM or present unintentionally, dye gradually accumulated in the lower chamber. Examples of various leaks on dye accumulation in the lower chamber are illustrated in Fig. 1B.

**Figure 2:**
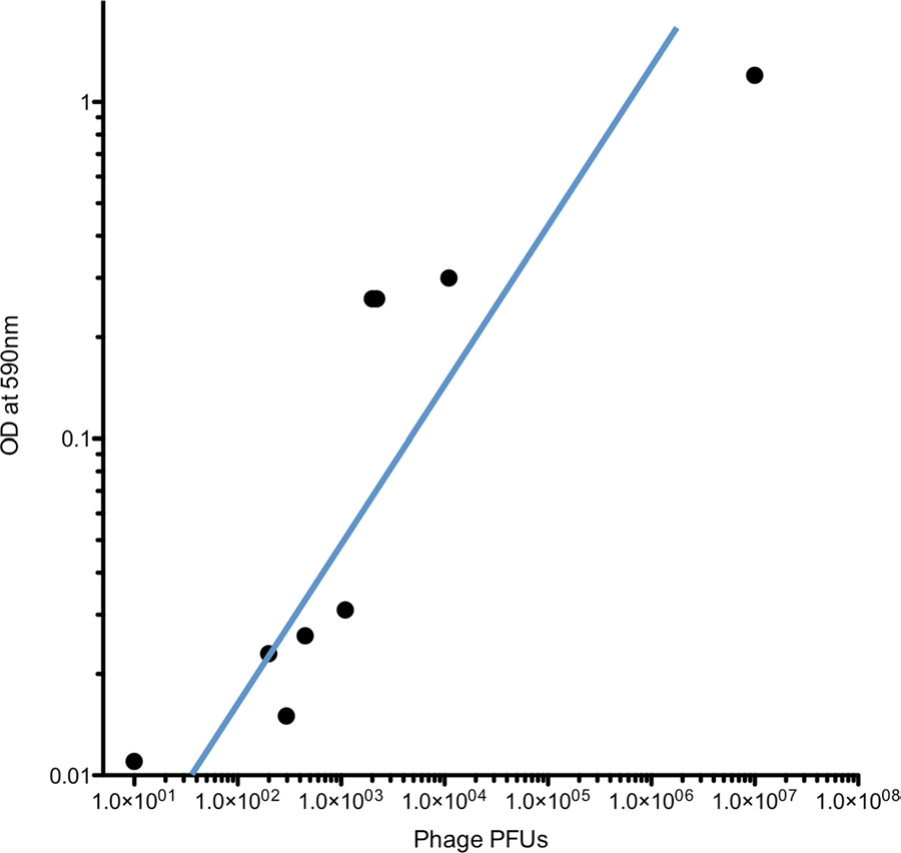
The relationship between trypan-blue dye detected in the lower chamber and transit of WT phage not bearing a peptide. Individual data are shown. The best-fit regression indicates that using trypan-blue OD at 590 nm as indicator of leaking in the chamber assay is both reliable and sensitive.

We also studied the titers of WT phage that were detected in the lower chamber, when leaks were induced in the TM fragment. In all of these WT phage experiments, trypan-blue dye was included in the upper chamber for leak detection. This allowed us to compare phage titers relative to dye levels. The intersect of this analysis, illustrated in Fig. 2, indicates that dye was able to detect leaks that resulted in approximately 30 phage particles. We have previously found titers of 0–100 WT phage to be the lower level of detection for WT phage presence in MEs after incubation of WT phage on the TM *in vivo*^31^. This level was presumed to represent the contamination threshold. Thus, the sensitivity of dye for leak detection was easily sufficient for use in the *in vitro* assay.

**Figure 3:**
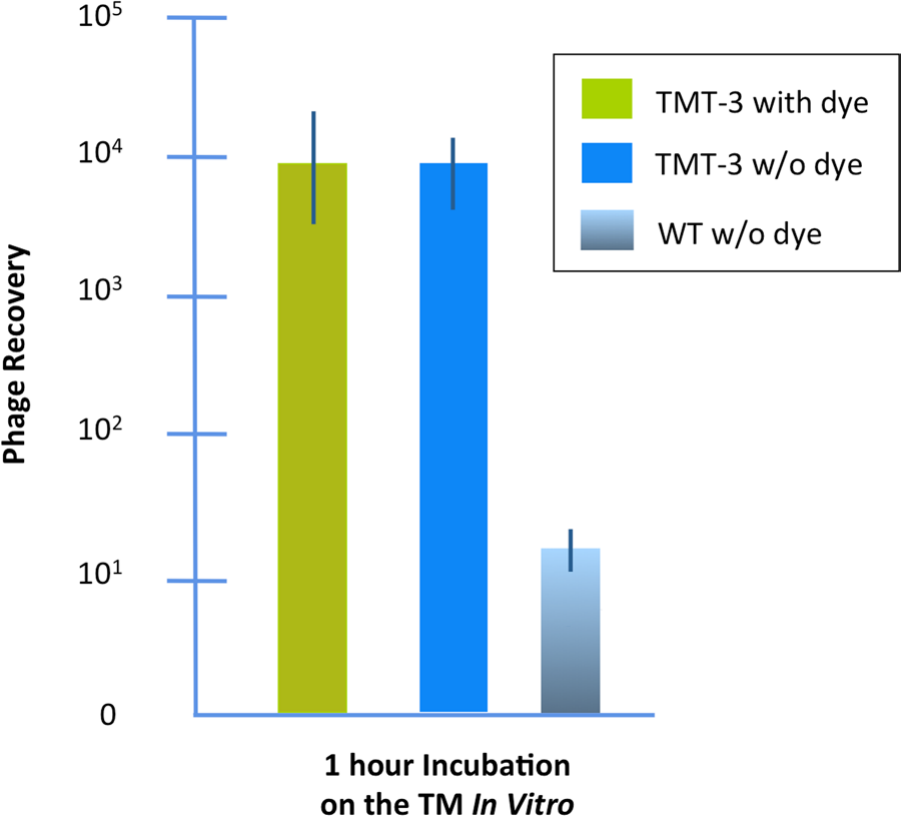
Transport of peptide phage TMT-3 with and without trypan-blue dye in the upper chamber. The dye had no apparent effect on trans-TM transport. Control is WT phage, not bearing a peptide, applied to the TM without dye.

We evaluated whether the presence of the dye in the upper chamber influenced the ability of a peptide phage known to actively cross the TM *in vivo*^31,32^, to transit an intact membrane fragment in the chamber. As can be seen from Fig. 3, there was no effect of dye on the transit of phage bearing peptide TMT-3 (SADSTKTTHLTL). This indicated that the trypan-blue leak detection method could be utilized during experimental tests of phage transit in the device. It was consequently used in all further experiments described below. Data from any experiment in which dye was noted in the lower chamber were discarded.

### Transit of peptide-bearing phage across the infected rat TM *in vivo* and *in vitro*

**Figure 4:**
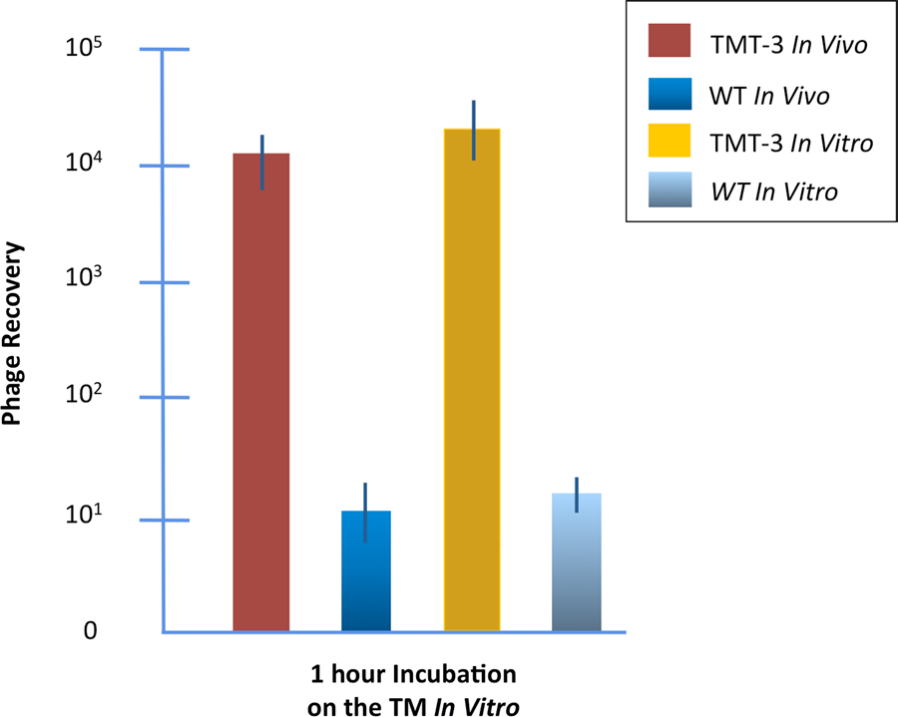
Transport of TMT-3 through the TM of rats *in vivo* and *in vitro*. The results of this study demonstrate that our *in vitro* assay with freshly harvested TMs is able to replicate trans-TM transport as it occurs *in vivo*. Controls are WT phage.

TM-transiting phage were identified in our prior studies by the ability to cross the infected rat TM. To evaluate transit in the *in vitro* assay, we compared the recovery of phage clone TMT-3, bearing the peptide SADSTKTTHLTL *in vitro* versus *in vivo* (Fig. 4). There was no significant difference in TMT-3 titer between the ME *in vivo* and the lower chamber *in vitro* after a 1-hr incubation on the exterior TM surface.

### Transit of peptide phage through the uninfected TM

**Figure 5:**
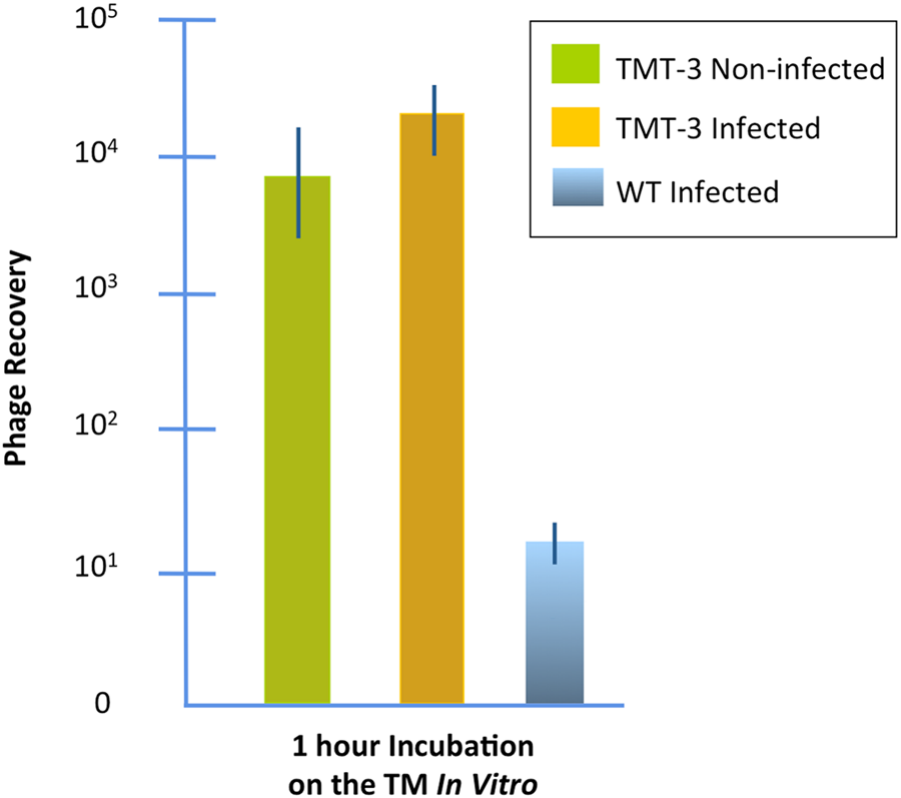
Transport of TMT-3 though the infected versus normal rat TM. These results demonstrate that the phenomenon of active trans-TM transport is not limited to the infected ME. Both previously infected and normal rat TMs exhibited transport. Control is WT phage on the infected TM.

To determine whether phage would cross the normal TM, in the absence of inflammation and other changes induced by ME infection, we compared the transit of TMT-3 phage across TM fragments from rats previously infected with NTHi with transit across TM fragments from uninfected rats. As can be seen in Fig. 5, transit was comparable between the two conditions, indicating that infection does not influence the transport mechanism.

### Transit of free peptide versus peptide phage thought the TM *in vitro*

**Figure 6:**
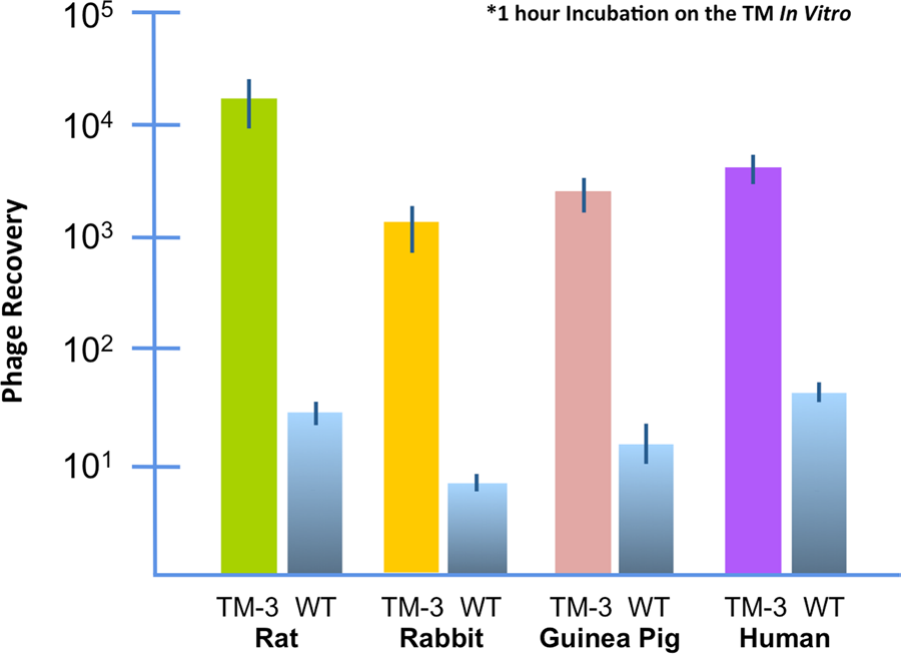
Transport of TMT-3 through the TM fragments for uninfected rats, guinea pigs, rabbits and humans *in vitro*, after a 1 hour incubation. We wished to determine whether trans-TM transport was limited to the rat, or was more widely shared among mammalian species including humans. Our data using the *in vitro* chamber suggest that trans-TM transport is widespread among mammals. Controls for each species were WT phage.

To determine whether peptide would be transported independent of phage, we compared recovery from the lower chamber of the assay after exposure of previously infected rat TM fragments to TMT-3 peptide phage, or free TMT-3 peptide linked to a PCR DNA-template, (Fig. 6). Peptide phage and peptide were similarly transported, while no transport of scrambled phage was observed.

### Transit of phage across the TMs of other mammalian species

**Figure 7:**
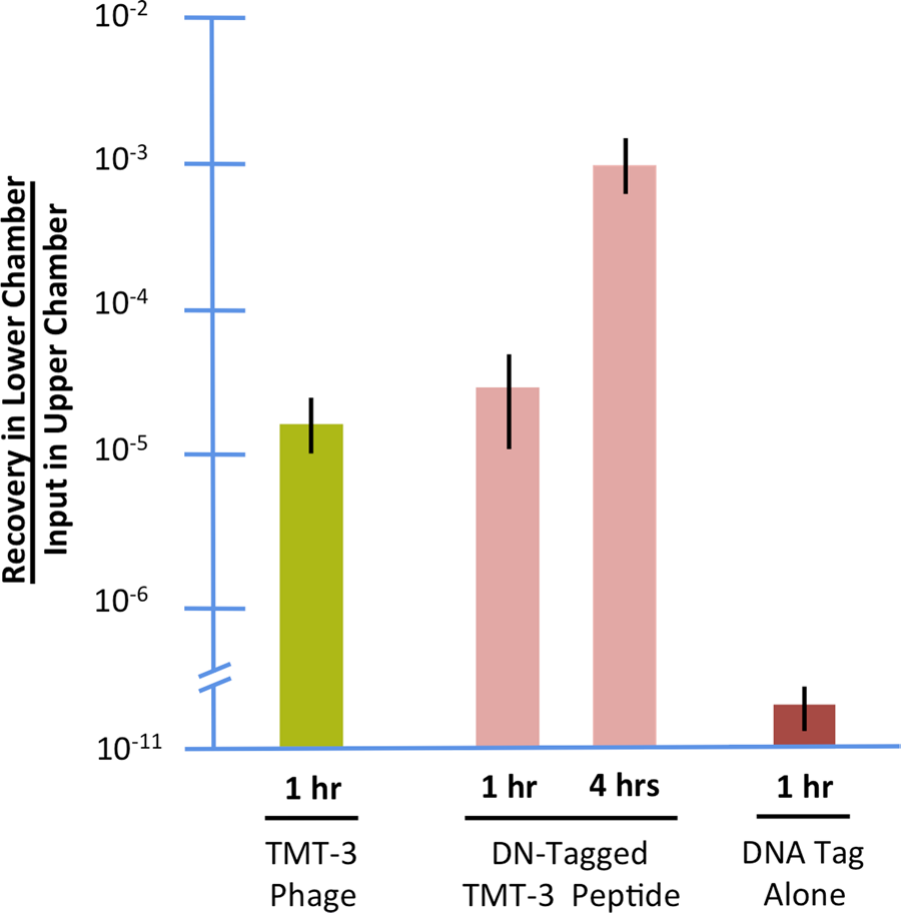
*In vitro* transport of TMT-3 peptide phage versus free peptide through TM fragments from previously infected rats. The amount of recovery of the phage-peptide vs. DNA-tagged peptide in the lower chamber relative to the concentration of material applied on top of the TM is reported. Transport occurred at similar levels across time. When the DNA tag alone was used as a control, detection in the lower chamber was at the detection threshold of the method, indicating virtually no transport.

We compared the ability of TMT-3 phage to cross the uninfected TMs of rat, guinea pig, and rabbit. TMT-3 was readily able to cross the TMs of all three species. Given this result, we then evaluated the ability of TMT-3 phage to cross fragments of the human TM that had been discarded after otologic surgery. Again, similar transport was observed (Fig. 7).

## Discussion

A major motivation for the development of our chamber assay was to determine whether TM fragments could be used *in vitro* to test trans-TM transport of peptides, since *in vivo* testing of human TMs is not possible. The results of this study validate the *in vitro* assay for brief post-mortem periods. Our prior observation that transport was observed through the *ex vivo* TM for approximately 6 hrs after death, but not for longer periods^31^, indicates that the assay can be used for at least a few hours after harvest of a TM fragment. The results also demonstrate that the chamber is able to hermetically isolate the external from the internal surfaces of TM fragments, as indicated by the failure of WT bacteriophage, not expressing a trans-TM peptide, to cross the TM and the exclusion of trypan-blue dye from the lower chamber when a leak or puncture is not present. The observation that *in vitro* and *in vivo* rat TM transport rates were similar presumably reflects the fact that the TM fragments used *in vitro* were not much smaller than the intact TM.

Another unanswered question was whether the phenomenon of active transport across the TM was limited to infected MEs, in which it was discovered, or whether the normal TM would also display transport. Our results determined that trans-TM transport occurs across the uninfected TM at levels similar to that seen after ME infection. Thus, the transport is not a characteristic of inflammation or other changes that occur during OM, but rather appears to be an innate characteristic of the membrane. The physiological purpose served by transport mechanism remains unknown.

**Figure 8:**
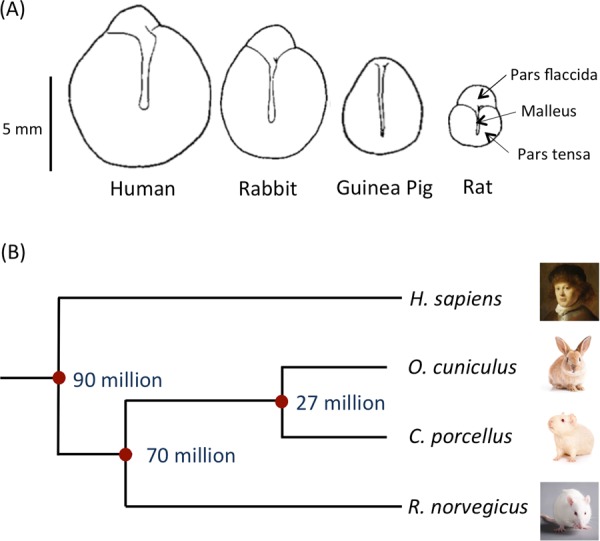
(**A**) Diagrams of tympanic membranes from the different species studied in this investigation showing relative sizes and shapes (adapted^36^). (**B**) Estimations of the time of divergence for the four species studied based on orthology relationship analyses using www.timetree.org. Images: **©** Shutterstock.

We also wished to determine whether trans-TM transport was limited to the rat, or was more widely shared among mammals. If the latter, the assay could be used to test human TMs. Our data suggest that trans-TM transport is widespread in mammals, occurring in suborders Myomorpha and Hystrichomorpha of the order Rodenta, as well as in the order Lagomorpha. These suborders are separated by many millions of years of evolution (Fig. 8).

The divergence of mammalian species has been extensively studied, both from the fossil record and more recently by the use of the “molecular clock” that estimates times of divergence based on mutations observed between the genomes of different species^33^. These analyses have been complicated by the sparseness of the early mammalian fossil record, and by potential differences in mutation rates after extinction events such as that at the K-T boundary^34^. However, it is generally thought that the divergence of the hystricomorph rodents including the guinea pig from the lagomorphs including the rabbit occurred 25–30 million years ago. The divergence of the hystricomoprh/lagomorph common ancestor from the myomorph rodents including rats and mice occurred approximately 60–80 million years ago^35^. Estimates for the divergence of the human ancestor from that of the lagomorphs and rodents range similarly from 65 to 130 years before the present^35^, but based on phylogeny it seems rather likely that this split occurred at least several million years before that of the Lagomorpha/Rodenta split, and probably fairly early in the radiation of placental mammals. Given the unlikeliness that trans-TM transport evolved independently in the species studied here, our data suggest that the mechanism by which peptides actively cross the TM is a very ancient trait. This in turn implies that the trait could be shared by many other mammalian species, including humans.

This conclusion was tested with fragments of human TM obtained during TM reconstructive and other otologic surgeries. We found that transport across human TM fragments was comparable to that seen for the fragments of TMs from the other species tested. This result indicates that active trans-TM transport could be harnessed for drug delivery of drugs, gene therapy vectors or other therapeutic agents across the intact TM and into the ME.

As can be seen in Fig. 6, transport across the TMs of species other than the rat, at the constant area of the assay, was less efficient. This could be due to differences in the structure or thickness of the TM. The structure of the TM of the species is basically similar^36^. An outer layer of keratinized epithelial cells joined by tight junctions is continuous with but thinner than that of the epidermis. Structural layers of primarily collagen fibers contain extracellular spaces that would allow free diffusion of molecules and phage particles. The inner epithelial cells, connected by tight junctions, is continuous with the mucosal epithelium of the middle ear. Thickness differences, are determined primarily by the connective tissue layers. The thickness of the pars tensa of the rat TM is approximately 5 μm, that of the guinea pig 10 μm and that of the rabbit 25 μm, as measured histologically^36^. The thickness of the human pars tensa as measured histologically is 35 μm^36^, while optical coherence tomography gives a thickness of 80–100 μm^37^. A comparison of these values to the data of Fig. 6 suggests that thickness is likely not a determining factor in transport rate.

Alternatively, the peptides discovered in the rat may not be optimal for interaction with the substrates of transport in the other species. It may be that different peptides are required for optimal transport in different species. Peptide library screening using human TM fragments could address this issue. Regardless, the larger size of the TM in the other species suggests that transport across their entire TMs *in vivo* might be roughly equivalent to that seen in the *in vivo* rat.

Another important factor for utilization of this transport mechanism is whether the presence of bacteriophage is required for trans-TM transport. We found that free peptides linked to a DNA tag crossed the TM as effectively as peptides attached to phage. This indicates that it is the peptide itself that determines trans-TM transport, not some combination of the peptide and phage amino acid sequence or properties. This result also suggests that peptides alone would be able to mediate transport of therapeutic agents into the ME. The characteristics of the peptides that mediate trans-TM transport are not known. However, we previously identified nine 12-mer peptides that are actively transported across the intact TM^31^, out of an original phage library content of 10^10^ different peptides. This suggests that very few other peptides share this characteristic. The transporting peptides are not similar to other cell or tissue-penetrating peptides, indicating that the mechanism by which they act is distinct.

It should be noted that all experiments were performed with the TM directly below the fluid containing the phage or peptide. While it is possible that gravity played a role in trans-TM transport, this seems unlikely. Active transport is more dependent upon the interaction of specific ligands with target molecules. Since peptides/DNA conjugates alone are transported efficiently, gravity settling of these molecules seems unlikely to have been significant within the time periods tested.

The fact that peptides and the much larger peptide-phage complex transit the TM at equivalent rates also has implications for the transport mechanism. Transport via a channel or transporter would presumably be far less efficient for the much larger peptide/phage particle. Similarly, paracellular transport^38^ by modification of tight junctions might also show a dependence on size. On the other hand, transcytosis^39^, mediated by endosomal vesicle transport, would likely be less influenced by size. Transcytosis has previously been recognized as a potential mechanism for transport of drugs and large particles across polarized cell layers.e.g.^40^^.^
